# The Hormonal Control of Food Intake

**DOI:** 10.1016/j.cell.2007.04.001

**Published:** 2007-04-20

**Authors:** Anthony P. Coll, I. Sadaf Farooqi, Stephen O'Rahilly

**Affiliations:** 1 Department of Clinical Biochemistry, Cambridge Institute for Medical Research, Addenbrooke's Hospital, Cambridge CB2 2XY, United Kingdom

## Abstract

Numerous circulating peptides and steroids produced in the body influence appetite through their actions on the hypothalamus, the brain stem, and the autonomic nervous system. These hormones come from three major sites—fat cells, the gastrointestinal tract, and the pancreas. In this Review we provide a synthesis of recent evidence concerning the actions of these hormones on food intake.

## Main Text

Despite marked day-to-day fluctuations in food intake and physical activity, most healthy adult mammals maintain a steady body weight over many years. This indicates that cumulative energy intake is normally matched with great precision to energy expenditure. It has long been proposed that homeostatic regulators control energy balance. In the mid 20th century, Gordon Kennedy was the first to propose that circulating signals generated in proportion to body fat stores influenced food intake and energy expenditure in a coordinated manner to regulate body weight (Kennedy, 1953). A series of contemporaneous experiments in which regions of the hypothalamus were physically ablated in rats showed that the hypothalamus was critical in this regulation (King, 2006). For example, damage to the ventromedial hypothalamus caused hyperphagia (eating in excess) and obesity whereas destruction of the lateral hypothalamus resulted in profound anorexia and weight loss. It was shown that circulating substances were capable of regulating food intake in parabiosis experiments (the surgical union of two animals allowing exchange of blood). Rats with lesions to the ventromedial hypothalamus parabiosed to normal animals remained hyperphagic and obese whereas the parabiosed partner ate less and lost weight. These experiments were the first to provide substantive evidence that a blood-borne satiety factor that was produced in obese animals required an intact hypothalamus for its activity.

Despite the robustness of these physiological observations it took more than 40 years before the molecules involved in such homeostatic control were identified. In the past decade the body of knowledge has grown rapidly and has resulted in a sea change in our understanding of the physiological processes that control food intake. We now know that a number of circulating peptides and steroids that are produced in the body can have a substantial influence on appetitive behavior through their actions on the hypothalamus, the brain stem, or afferent autonomic nerves. These hormones come from at least three sites: fat cells, the gastrointestinal tract, and the endocrine pancreas.

### Leptin

Progress toward identifying a circulating compound regulating satiation came from studying two naturally occurring obese mouse models, *obese* (*ob*/*ob*) and *diabetic* (*db*/*db*). Parabiosis experiments revealed *ob*/*ob* mice were deficient in a circulating satiety factor, whereas *db*/*db* mice produced the factor in excess but lacked the ability to respond to it (see Friedman and Halaas, 1998). *ob*/*ob* mice were shown to harbor a loss-of-function mutation in the gene encoding a secreted peptide, named leptin, that is produced largely, possibly exclusively, in adipose tissue (Zhang et al., 1994). Circulating concentrations of leptin appeared to mirror fat cell stores—increasing with overfeeding and decreasing with starvation. Leptin administration to obese leptin-deficient animals reversed their hyperphagia, hypothermia, decreased locomotor activity, and all neuroendocrine and immunological abnormalities (Friedman and Halaas, 1998). Humans genetically lacking leptin are also hyperphagic and severely obese and respond dramatically to leptin administration (Friedman and Halaas, 1998). Administration of leptin to fasted mice abrogates many of the neuroendocrine consequences of starvation, suggesting that the normal biological role of leptin may be to aid the organism in monitoring transitions between adequately nourished and starved states (Friedman and Halaas, 1998).

The *db*/*db* phenotype is due to mutations in the receptor for leptin (OB-R), a class 1 cytokine receptor. A variety of homozygous and compound heterozygous loss-of-function mutations in the leptin receptor have also been found in severely obese humans (Farooqi et al., 2007). There are at least six isoforms of the leptin receptor (OB-Ra-f), primarily as a consequence of alternate splicing. The main signaling isoform (and longest) is Ob-Rb. It is highly expressed in the hypothalamus and on immune cells and signals via the JAK-STAT pathway. Although the shorter forms of the receptor are more widely expressed than OB-Rb, their precise functions are unknown.

Direct administration of leptin into the central nervous system (CNS) of *ob*/*ob* mice in doses that do not result in detectable levels in the periphery completely reverses the body weight and metabolic phenotype (Friedman and Halaas, 1998), emphasizing the importance of these central leptin receptors in most actions of leptin. Although leptin is effective at reducing food intake and body fat in leptin-deficient and normal rodents, it is relatively ineffective in doing so in mice with diet-induced obesity. This is in accord with the observations that in human populations plasma leptin is positively correlated with fat mass; leptin administration to obese humans only has a modest and variable effect on body weight. These observations have led to the concept that “leptin resistance” may underlie the failure to regulate energy stores as seen in common forms of obesity. Although leptin has not become the panacea for obesity that was once hoped, it has undoubted actual and potential clinical utility. It is life-saving in humans with congenital leptin deficiency (Farooqi et al., 2002), has dramatic effects on the metabolic state of patients with lipodystrophy (a metabolic disorder characterized by selective loss of body fat and low leptin levels) (Oral et al., 2002), and shows promise as a therapeutic/diagnostic agent for weight-related reproductive disorders (Welt et al., 2004). Finally, up to 10% of obese people have relatively low leptin levels. Therapeutic trials of leptin supplementation in these individuals are overdue when, in combination with other agents, such therapy might be well suited to prevent weight regain after weight loss.

The critical importance of leptin in the control of energy homeostasis has been clearly established. However multiple intriguing questions remain. How and where does leptin act in the brain to regulate food intake and metabolism? What molecular mechanisms underlie leptin resistance? How is leptin secretion regulated? And what determines the large variation in leptin levels among individuals?

#### Leptin-Responsive Neurons in the Brain

**Figure 1:**
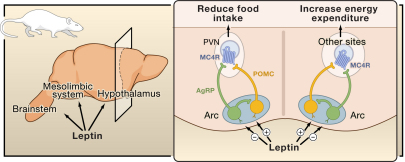
Control of Food Intake by the Hypothalamic Leptin-Melanocortin Pathway The hypothalamus receives and integrates neural, metabolic, and hormonal signals to regulate energy homeostasis. In particular, the adipocyte-derived hormone leptin and the melanocortin pathway have a critical role in the control of food intake. AgRP, Agouti-related protein; Arc, arcuate nucleus; MC4R, melanocortin 4 receptor; POMC, pro-opiomelanocortin; PVN, paraventricular nucleus.

The hypothalamus receives and integrates neural, metabolic, and humoral signals from the periphery. In particular, contained within the arcuate nucleus of the hypothalamus are two populations of cells that are the best characterized leptin-responsive neurons in the brain (Cone, 2005). The first population of neurons express two potent appetite-stimulating (orexigenic) peptides, the melanocortin anatagonist Agouti-related peptide (AgRP) and Neuropeptide Y (NPY). The second population expresses the peptide cocaine and amphetamine-related transcript (CART) and the large precursor peptide pro-opiomelanocortin (POMC). Both sets of neurons project to second-order, melanocortin 4 receptor (MC4R) expressing neurons within the hypothalamus and elsewhere in the brain (Figure 1).

Leptin inhibits NPY/AgRP neurons, and fasting significantly upregulates the expression of NPY and AgRP. Interestingly, germline deletion of NPY or AgRP has no major effect on body weight (see Cone, 2005; Flier, 2004), although mice lacking AgRP do become modestly lean late on in life due to an increase in energy expenditure (Wortley et al., 2005a). Recently four independent groups, each using slightly different strategies, genetically ablated NPY/AgRP neurons postnatally (Flier, 2006). Although the extent to which NPY/AgRP neurons were ablated in each study differed considerably, the message was clear—loss of NPY/AgRP neurons in adult life leads to profound, life-threatening hypophagia. Interestingly, when these neurons are ablated in the newborn period the effects on body weight and food intake are much more modest, suggesting that network-based compensatory mechanisms can develop in neonates but do not readily occur in adults. It is as yet unclear whether the effects of ablation of these neurons on food intake are solely due to the loss of NPY and AgRP or whether the loss of other neurotransmitters is involved.

In contrast to NPY/AgRP neurons, POMC/CART neurons are stimulated by leptin and fasting decreases POMC expression. Although CART deficiency does not appear to influence energy balance, there is clear evidence that POMC peptides play a critical role in feeding behavior with both POMC-deficient mice and humans developing hyperphagia and obesity (Coll et al., 2004). POMC undergoes extensive posttranslational modification to generate a range of smaller biologically active peptides, the melanocortins, which are agonists for melanocortin receptors. The ultimate pool of bioactive melanocortins released from POMC-expressing neurons is influenced by a number of factors including the activities of prohormone convertases, carboxypeptidases, and both acetylases and deacetylases (Coll et al., 2004). Although α-melanocyte-stimulating hormone (α-MSH) has always been considered the predominant POMC-derived product controlling energy balance, recent genetic evidence strongly implicates β-MSH in appetite regulation, at least in humans (Biebermann et al., 2006; Lee et al., 2006).

Leptin receptors are also found outside of the arcuate nucleus, although the importance of leptin's activity at these other sites has not been clear until recently. For instance, although the selective ablation of leptin receptors in POMC neurons of the arcuate nucleus results in obesity (Balthasar et al., 2004), it is less pronounced than in mice that globally lack leptin receptor. Indeed, there are now clear data to show that leptin receptors on other neurons, such as those that express steroidogenic factor 1 (SF1) in the ventromedial hypothalamus, are required for normal body weight (Dhillon et al., 2006). Mice lacking leptin receptors on these neurons in the ventromedial hypothalamus become obese, despite having no discernible increase in food intake. Interestingly, when placed on a high-fat diet, these mice appear unable to suppress food intake and adequately stimulate energy expenditure. Thus leptin receptors in SF-1 neurons of the ventromedial hypothalamus may play an important role in the adaptive changes critical for resisting diet-induced obesity.

However, there is still more to learn. Although mice with a combined deficiency of leptin receptors on both SF-1 and POMC neurons weighed more than those with loss of either alone, they were still less obese than mice that globally lack leptin receptors, indicating that there must be other sites important for leptin action. Two such sites may be the caudal brainstem and the ventral tegmental area in the midbrain. The caudal brainstem contains both leptin-responsive, Ob-Rb-expressing neurons (Grill et al., 2002) and a population of POMC neurons, just like the arcuate nucleus. However, there appear to be some intriguing and important differences between the brainstem and the arcuate nucleus.

Although fasting induced a fall in POMC mRNA in both regions, in contrast to the arcuate, the reduction seen in the brainstem was not reversed by leptin administration. Furthermore, leptin did not cause STAT-3 phosphorylation or c-*fos* activation within brainstem POMC neurons, suggesting that leptin signaling via POMC-derived peptides in the CNS occurs entirely via hypothalamic POMC neurons (Huo et al., 2006).

The ventral tegmental area contains dopaminergic neurons that have long been thought to play a role in the reward and motivational aspects of ingestive behavior. Two recent studies now clearly indicate that by acting upon these dopaminergic neurons, leptin influences food consumption in a hitherto unrecognized manner (Fulton et al., 2006; Hommel et al., 2006).

### Further Downstream of Leptin

#### Melanocortin Receptors

Genetic deletion of MC4R in mice and humans results in severe hyperhagic obesity (Coll et al., 2004). MC4R mutations are responsible for up to 5% of cases of severe childhood obesity and between 0.5% and 2.5% of adult obesity (Hinney et al., 2006; Larsen et al., 2005). Estimates of the frequency in the general population in the UK suggest a mutational frequency of 1/1000 (Alharbi et al., 2007), making MC4R deficiency one of the most common single-gene disorders. The phenotypic features of MC4R deficiency include hyperphagia, an increase in fat and lean mass, and an increase in bone mineral density (Farooqi et al., 2003). Of particular note is the finding that the severity of receptor dysfunction seen in in vitro assays can predict the amount of food ingested at a test meal by the subject harboring that particular mutation (Farooqi et al., 2003).

Until recently, it was uncertain as to whether each feature of the complex phenotype of MC4R deficiency could be ascribed to different regions of the brain. Did one site control food intake whereas another controlled energy expenditure? There are now clear data demonstrating that there is indeed functional divergence of the melanocortin pathway (Balthasar et al., 2005). Using *cre-lox* technology, Balthasar and colleagues partially “rescued” MC4R-deficient mice by re-expressing *Mc4r* only in the paraventricular hypothalamus and the amygdala. Although still heavier than control littermates, these mice were significantly lighter than mice globally lacking MC4Rs. This was entirely due to a normalization in food intake because the reduced energy intake typical of mice globally lacking *Mc4r* was unaffected by this targeted re-expression. Thus MC4Rs in the paraventricular hypothalamus and/or amygdala appear to control food intake, but MC4Rs expressed elsewhere have a role in the control of energy expenditure.

The data that define the physiological function of MC3R in energy metabolism are not as dramatic as those for MC4R, although it is likely these two receptors serve nonredundant roles (Coll et al., 2004) Mice lacking *Mc3r* have a unique phenotype—despite an increased fat mass, their total body weight is similar to wild-type mice due to a reduction in lean mass. MC3R may influence feed efficiency (weight gain per kcal consumed) and the partitioning of fuel stores into fat.

#### BDNF/TrkB

A number of recent studies have given insights into the mediators and signaling mechanisms that lie beyond the melanocortin receptors. In particular, a growing body of evidence implicates brain-derived neurotrophic factor (BDNF) as a player in energy homeostasis. BDNF is a regulator of brain development and plasticity and exerts its effects through the tyrosine kinase receptor TrkB. Both BDNF and its receptor TrkB are widely expressed in the brain. Mice lacking either one copy of the *Bdnf* gene or with a tissue-specific conditional deletion of *Bdnf* in the postnatal brain develop hyperphagia and obesity (Rios et al., 2001). Administration of BDNF can ameliorate the hyperphagia and metabolic disturbance in *db*/*db* mice (Nakagawa et al., 2000). An exciting study reported that MC4R and BDNF/TRk-B-mediated signaling may be coupled in the regulation of energy balance (Xu et al., 2003). Xu et al. reported that BDNF was highly expressed in the ventromedial nucleus, with expression regulated by nutritional status and by MC4R signaling. The authors also showed that mice with a hypomorphic mutation in *trkb* (resulting in expression levels 25% of normal) closely resembled mice lacking *Mc4r* in that they developed hyperphagia and obesity, increased body length, and excessive weight gain on a high-fat diet. Further, central infusion of BDNF into mice with deficient MC4R signaling suppressed the hyperphagia and excessive weight gain observed on high-fat diets. In humans, genetic disruption of the neurotrophin receptor TrkB (Yeo et al., 2004) and in its ligand BDNF (Gray et al., 2006) cause severe hyperphagia and obesity, developmental delay, impaired short-term memory, and unusually hyperactive behavior.

#### Melanin-Concentrating Hormone

Melanin-concentrating hormone (MCH) is an orexigenic (appetite-stimulating) peptide produced by neurons in the lateral hypothalamus. Robust data from rodent models demonstrate that MCH has a critical role in energy balance (Flier, 2004). Mice lacking MCH are hypophagic and lean whereas transgenic overexpression of MCH in the lateral hypothalamus leads to obesity and insulin resistance. The key role of this neuropeptide in integrating the downstream effects of leptin is demonstrated by the fact that MCH expression is increased in hypothalami of *ob*/*ob* mice, with a dramatic reduction in body fat seen in *ob*/*ob* mice also lacking MCH.

Of note, loss of the receptor believed to be responsible for the action of MCH (MCH1R) results in hyperphagia, but mice lacking *Mch1r* mice remain lean as a consequence of hyperactivity and altered metabolism. More recently, targeted ablation of MCH neurons has been demonstrated to cause hypophagia, increased energy expenditure, and late-onset leanness (Alon and Friedman, 2006).

#### Orexins

Orexins were originally identified as peptides produced selectively in the lateral hypothalamus (Sakurai et al., 1998). Central administration of orexin appeared to increase food intake in mice leading to the initial view that the principal function of orexins was the control of food intake. However, subsequent studies suggest that orexins play a more important role in the maintenance of alertness with genetic or acquired deficiency of orexin signaling resulting in narcolepsy (Saper et al., 2005). A possible link with the leptin and the adipostatic pathways remains in that leptin administration decreases orexin expression whereas fasting increases *orexin* mRNA levels (Yamanaka et al., 2003).

#### Others

The hypothalamic transcription factor Single-minded 1 *(Sim1*) is expressed in a number of regions known to be involved in energy homeostasis, including the paraventricular nucleus and the lateral hypothalamus. Haploinsufficiency of *Sim1* is associated with hyperphagic obesity and increased linear growth (Kublaoui et al., 2006; Michaud et al., 2001), closely resembling the phenotype of agouti yellow (A*y*) and *Mc4r* null mice, two classic models of disrupted hypothalamic melanocortin signaling. These similarities may be because MC4 receptors involved in the regulation of food intake signal through Sim1 and/or its transcriptional targets. *Sim1* heterozygous mice remain hyperphagic despite elevated hypothalamic *Pomc* expression and have an impaired anorectic (appetite-suppressing) response to melanocortins, suggesting that *Sim1*-expressing neurons in the paraventricular nucleus regulate feeding in response to melanocortin signaling (Kublaoui et al., 2006).

Serotonin (5-hydroxytrypamine, 5-HT) derived drugs have long been known to have clinical utility in the induction of weight loss, but the neural pathways through which central serotonergic systems regulate food intake have remained elusive. It now appears that serotonin-induced hypophagia requires downstream activation of MC4R, with the central melanocortin system a key site of action of 5-HT. Not only does 5-HT inhibit NPY/AgRP neurons but it also activates POMC neurons, leading to an increase in activity at MC4R in target sites and a reduction in food intake (Heisler et al., 2006).

### Developmental Effects of Leptin

Recent data have elegantly described how leptin can potently influence neuronal growth and development. By studying the electrophysiological activity in neurons from *ob*/*ob* mice, it was shown that leptin deficiency markedly changes synaptic inputs to arcuate neurons, increasing excitatory inputs on NPY/AgRP neurons but decreasing excitatory inputs to POMC neurons (Pinto et al., 2004). Critically, within hours of administration to *ob*/*ob* mice, leptin was able to reverse these effects, thereby demonstrating it to be a crucial regulator of synaptic plasticity. Leptin appears also to be a neurotrophic growth factor during hypothalamic development (Bouret et al., 2004) with leptin deficiency severely reducing the density of innervations from the arcuate nucleus to the paraventricular nucleus, lateral hypothalamus, and dorsomedial nucleus—all regions implicated in the control of energy balance. Leptin replacement in adult life appears ineffective in reversing this axonal pattern, but, in sharp contrast, replacement in early postnatal life (a time when a surge in plasma leptin levels in rodents has long been recognized) can restore the density of innervations to second-order neurons to that of wild-type mice. This raises interesting questions as to whether there is a critical period of leptin-dependent hypothalamic development in the early neonatal period that may have major ramifications for energy balance later in life. If true, this “altered wiring” could, in part, explain the profound, early-onset obesity seen in leptin-deficient children.

However, the fact that leptin replacement therapy in leptin-deficient adults with established morbid obesity results in profound weight loss (Licinio et al., 2004) means that even if a hypothalamic signaling system developed entirely in the absence of leptin, the ability to respond in a physiological manner to leptin is retained. Further, studies using the neurocytokine ciliary neurotrophic factor (CNTF) suggest that a previously unappreciated degree of neuroproliferative potential continues into adult life (Kokoeva et al., 2005). CNTF has the remarkable ability to induce weight loss that persists after the cessation of treatment. CNTF induces cell proliferation in feeding centers within the hypothalamus, with many of the newborn cells behaving like neuronal cells known to be critical in energy balance.

### Mechanisms of Leptin Resistance

In the majority of cases of obesity, despite both an intact leptin receptor and high circulating levels, leptin fails to bring about weight loss. This diminished response to the anorexigenic effects of leptin is referred to as “leptin resistance.” What may be the potential mechanisms underlying this resistance?

A failure of circulating leptin to reach its target receptor within the brain appears to be one mechanism at work in rodents with diet-induced obesity, a classic model of leptin resistance. In contrast, leptin resistance in aged rats may result from decreased expression of the leptin receptor within hypothalamic neurons (see Bates and Myers, 2003; Flier, 2004). However, a hypothesis that has received recent attention is that leptin resistance may be due to an attenuation of the intracellular leptin signaling cascade. Suppressor of cytokine signaling-3 (Socs-3) is an intracellular protein that negatively regulates the action of various cytokine receptors. It is now recognized to act to limit leptin signaling and therefore is a potential mediator of leptin resistance. Indeed, although complete lack of *Socs-3* is embryonic lethal, mice with only one functional copy of *Socs-3* or those with neuronal deletion of *Socs-3* are lean and leptin sensitive (Howard et al., 2004; Kievit et al., 2006; Mori et al., 2004). Kievit et al. further highlighted the key role Socs-3 plays in leptin signaling by demonstrating that selective deletion of Socs-3 from POMC-expressing neurons alone also results in enhanced leptin sensitivity and resistance to weight gain on a high-fat diet. Finally, recent data that demonstrate that mice with a neuron-specific lack of the tyrosine phosphatase PTP1B are rendered hypersensitive to leptin point to this molecule having an important role in the development of leptin resistance (Bence et al., 2006).

Leptin resistance remains enigmatic. In part this is because of the inaccessibility of the relevant leptin-resistant neurons in humans and the difficulty of isolating unique neuronal subpopulations in a mass of other neurons presumably not involved in leptin action. In contrast to the history of insulin resistance when the characterization of the insulin receptor, combined with accessibility of insulin's target tissues, rapidly permitted the classification of insulin-resistant states as “pre-receptor,” “receptor-related,” or “post-receptor,” it is still extremely difficult to confidently classify leptin-resistant humans or rodents in this manner.

### What Regulates Leptin Secretion?

The adipocyte is not a classical endocrine cell and leptin is not stored in typical endocrine secretory granules. The amount of leptin produced by an adipocyte appears to be regulated at the transcriptional level but also at the levels of translation, storage, turnover, and secretion (Lee and Fried, 2006). Leptin levels do show some diurnal variation, but this appears to be entrained by meal times in rodents. Insulin and glucocorticoids positively regulate leptin production whereas agents that increase cAMP levels in the adipocyte, such as β adrenergic agonists, suppress leptin production (Ricci et al., 2005). The marked sexual dimorphism in plasma levels (much higher in females than males) is, at least in part, explained by a suppressive effect of androgens on leptin production. The precise mechanisms whereby increased fat stores are signaled to the adipose tissue mass to produce more leptin remains mysterious, and progress has been impeded by very low levels of leptin made in the otherwise very useful adipocyte cell lines in which much adipocyte cell biology has been established.

### Other Signals from Adipocytes

#### Adiponectin

Adiponectin is an adipocyte-derived hormone that has been proposed to play an important role in energy homeostasis (Trujillo and Scherer, 2005). It has strong sequence homology with C1q and type VIII and X collagen and its C-terminal globular domain has a tertiary structure that resembles TNFα. In sharp contrast to leptin, plasma adiponectin levels are negatively correlated with body fat, decreasing with obesity and increasing in response to weight loss. Adiponectin also circulates in plasma at a much higher concentration than leptin (μg/ml versus ng/ml, respectively). Data from knockout mouse models suggest that adiponectin is protective against the development of insulin resistance, glucose intolerance, and dyslipidemia (Nawrocki et al., 2006). However, the evidence that adiponectin has a role in controlling food intake is less convincing. Although ectopic overproduction of adiponectin can reduce food intake in high-fat-fed rats and offset the development of diet-induced obesity (Shklyaev et al., 2003), food intake in adiponectin knockout mice is no different from that seen in wild-type littermates (Maeda et al., 2002).

#### Interleukins

Interleukin 6 (IL-6) is a cytokine that has diverse roles in immunoregulation and the inflammatory response. It is also secreted from adipose tissue, particularly omental fat (the fat surrounding the bowels), independently of any acute inflammatory condition. Like leptin, IL-6 levels correlate with total body fat. Mice lacking IL-6 are not hyperphagic but become modestly obese in adult life as a result of disruption in energy expenditure (Wallenius et al., 2002). However, a combined deficiency of IL-6 and interleukin 1 (IL-1) does cause hyperphagia and a more marked obesity (Chida et al., 2006). In addition, loss of the fat-derived cytokine interleukin 18 (IL-18) leads to hyperphagia and obesity. Interestingly, the administration of recombinant IL-18 does not alleviate hyperphagia in IL-18 knockout mice if injected intravenously but does if it is injected into the cerebral ventricles (Netea et al., 2006). This suggests that IL-18 might have a role in the regulation of food intake by the CNS.

### The Gastrointestinal Tract

#### Cholecystokinin

**Figure 2:**
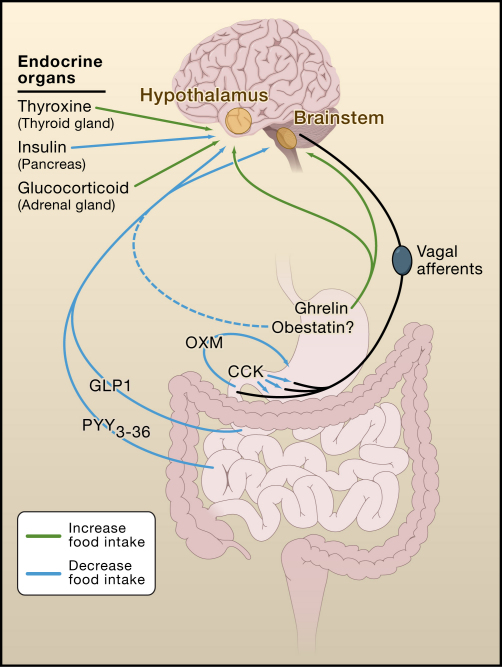
Hormones from the Gut and Endocrine Organs Affect Food Intake Hormonal signals derived from the gut and classical endocrine organs, such as the pancreas, thyroid, and adrenal glands act in synergy to effect changes in feeding behavior. CCK, cholecystokinin; OXM, oxyntomodulin; GLP-1, glucagon-like peptide 1; PYY_3–36_, peptide YY_3–36_.

Cholecystokinin (CCK) is a gut peptide that has long been established to act as a postprandial satiety signal (Chaudhri et al., 2006). It is released into the circulation from enteroendocrine cells of the duodenum and jejunum in response to fatty acids (Figure 2). CCK acts at receptors on peripheral vagal afferent terminals, which transmit signal to appetite centers, such as the nucleus of the solitary tract, contained within the brainstem. Peripheral administration of CCK also activates mouse POMC neurons in the nucleus of the solitary tracts with signaling via MC4Rs in this region appearing to be crucial in bringing about the satiety effects of CCK. This peptide is ineffective in reducing food intake in mice lacking MC4R and in mice in which brainstem melanocortin receptors are blocked pharmacologically (Fan et al., 2004). Thus in addition to integrating long-term adipostatic signals like leptin, the melanocortin system may also be important in integrating short-term gut-derived satiety signals.

#### PYY_3–36_

Peptide YY (PYY) is a 36 amino acid peptide secreted from the endocrine L cells of the gut. Circulating PYY levels are low in the fasting state and rapidly increase postprandially when two forms, PYY_1–36_ and PYY_3–36_, are released into the circulation. Both peptides have local effects on gut motility and both have the ability to increase food intake if administered directly into the cerebrospinal fluid of animals. In contrast, peripherally administered PYY_3–36_ can reduce food intake (Chaudhri et al., 2006) . Like leptin, the appetite-suppressing effects of PYY_3–36_ were initially thought to be mediated indirectly through the central melanocortin system. However, this appears not to be the case as a disrupted melancortinergic system still permits the full anorexigenic effects of PYY_3–36_ (Coll et al., 2004). Some groups have reported difficulty in reliably reproducing the anorexigenic effects of PYY_3–36_ (Tschop et al., 2004), a phenomenon that may reflect the influence of environmental stimuli on the ability of the animals to respond. It has been suggested that the inhibition of food intake by PYY_3–36_ is dependent, at least in part, on the induction of an aversive response (Halatchev and Cone, 2005). However, recent data from a new *Pyy* null mouse model have supplied evidence that PYY may indeed have a physiological role in eating behavior, in particular mediating the satiating effects of dietary protein (Batterham et al., 2006).

In humans, PYY_3–36_ levels are elevated in many disease states that are characterized by weight loss. Overweight subjects have been reported to have a relative deficiency of postprandial PYY_3–36_ release associated with reduced satiety (le Roux et al., 2006b) and bariatric surgery results in an exaggerated postprandial PYY_3–36_ surge, potentially explaining the effectiveness of such surgery in maintaining a prolonged reduction in postoperative weight (le Roux et al., 2006a). Whether or not PYY_3–36_ is a true endogenous physiological regulator of food intake, long-term trials of PYY_3–36_ as an antiobesity agent are ongoing, and the results are awaited with great interest.

#### Ghrelin

Ghrelin was discovered as an endogenous ligand for the growth hormone secretagogue receptor (GHSR). The octanoylated 28 amino acid peptide is produced and secreted by cells within the oxyntic glands of the stomach. Ghrelin mRNA expression and peptide secretion are increased by weight loss, fasting, and insulin-induced hypoglycaemia. Peripheral administration of ghrelin stimulates food intake and decreases fat utilization. Thus, ghrelin has been proposed to be an enteric signal involved in energy homeostasis, being unique in that it stimulates appetite rather than acting as a satiety signal (Williams and Cummings, 2005).

There is considerable evidence to indicate that the classical arcuate-based pathways are central to ghrelin's effects on food intake (Williams and Cummings, 2005). Given this, the first reports of mice with targeted deletions of ghrelin exhibiting no changes in feeding behavior or body composition were somewhat surprising (Sun et al., 2003; Wortley et al., 2004). GHSR knockout animals were also only modestly lean (Sun et al., 2004). However, more detailed studies of the metabolic phenotype of mice lacking ghrelin (Wortley et al., 2005b) or GHSR (Zigman et al., 2005) report that both are resistant to diet-induced obesity when fed a high-fat diet, eating less and preferentially utilizing more stored fat as an energy substrate than wild-type mice. These data lend support to the notion that ghrelin-responsive pathways are an important component of coordinated body-weight control. Very recently there has been a report that rats vaccinated with ghrelin can produce neutralizing antibodies to the hormone and that this is associated with decreased rates of weight gain (Zorrilla et al., 2006).

In addition to being produced in the stomach, ghrelin may also be produced within the brain. A new set of ghrelin-positive neurons within the hypothalamus, lying between the dorsal, ventral, paraventricular, and arcuate nuclei, were identified by immunohistochemistry (Cowley et al., 2003). The functional relevance of brain-derived ghrelin remains to be determined. However, there is now evidence that the central actions of ghrelin are of physiological relevance in the control of adipocyte metabolism (Theander-Carrillo et al., 2006). Chronic infusion of ghrelin into the CNS not only increases lipogenesis and inhibits lipid oxidation in white adipose tissue but also decreases expression of UCP 1 and 3 in brown fat, in keeping with a reduction in energy expenditure. Thus, central ghrelin appears to partition nutrients toward fat storage by favoring an increase in glucose and triglyceride uptake, increasing lipogenesis, and inhibiting lipid oxidation in white adipocytes. This fits in well with the phenotype of ghrelin-deficient mice described earlier, where under conditions of abundant dietary fat, the impaired adipocyte metabolism becomes functionally relevant and leads to a decreased susceptibility to diet-induced obesity. Further, this may also suggest an alternative role for the well-known pre-meal surge in ghrelin. Rather than being a signal of meal initiation, this change in circulating ghrelin may trigger in the CNS processes that prepare the body to receive and appropriately process in-coming nutrients.

#### Obestatin

Ghrelin is derived from the posttranslational processing of the 117 amino acid prohormone preproghrelin. A recent study by Zhang et al. has suggested that subtle but distinctly different processing of this precursor generates a peptide that directly opposes the effect of ghrelin on food intake (Zhang et al., 2005). Using a bioinformatics search to look for typical enzymatic cleavage sites in other large precursors of peptide hormones, Zhang et al. identified a 23 amino acid region of pre-proghrelin that is flanked by potential convertase cleavage sites. Further characterization of the predicted peptide revealed it to be a circulating peptide, which, like ghrelin, was also detectable within the stomach wall of rats. In vivo studies with a synthetic version of the peptide demonstrated that it suppressed food intake, inhibited jejeunal contraction, and decreased body weight, all in sharp contrast to the orexigenic effects of ghrelin. On the basis of these suppressive effects on food intake the peptide was duly named “obestatin”—derived from the Latin “obedere” —to devour. By screening a range of mammalian orphan receptors, Zhang et al. showed that obestatin appeared to be working via GPR39, a G protein-coupled receptor that is expressed in the stomach, in the intestines, and within the hypothalamus. Thus, it appeared that two antagonistic hormones were derived from the same gene, with differential posttranslational modification resulting in peptide products with apparently opposing effects. Certainly, this neat processing trick might be an attractive hypothesis to explain how models of ghrelin deficiency outlined earlier appear to have such a bland phenotype. In knocking out the orexigen ghrelin, mutant mice were also rendered deficient in the anorexigen obestatin. However, the true biological function of obestatin remains uncertain. For example, the circulating levels of obestatin appear to be far lower than those of ghrelin, and in sharp contrast to ghrelin, there appears to be no change in circulating obestatin upon fasting or re-feeding in rodents. Although these anomalies may be due to as yet unappreciated subtlety in the peptide-processing systems, the excitement of the initial report must be tempered by more recent data that have failed to show any effect of obestatin on food intake in rodents (Holst et al., 2007; Nogueiras et al., 2007).

#### Glucagon-like Peptide-1

Glucagon-like peptide-1 (GLP-1) is a peptide product of the proglucagon gene, released from the L cells of the small intestine in response to food ingestion (Drucker, 2006). GLP-1 is a potent inducer of glucose-dependent insulin release. This has lead to the development of GLP-1 agonists that have clinical utility in the treatment of type 2 diabetes mellitus (Drucker, 2006). GLP-1 can also influence food intake with the GLP-1 analog exenatide, capable of lowering both blood glucose and body weight in obese type 2 diabetic subjects. The effects on body weight may be as a result of induction of satiety via inhibition of gastric emptying, but there is also evidence that GLP-1 can influence feeding behavior by acting at the nucleus of the solitary tract in the brainstem and the paraventricular nucleus of the hypothalamus (Chaudhri et al., 2006).

#### Oxyntomodulin

Like GLP-1, oxyntomodulin (OXM) is also produced from the proglucagon gene and released from the small intestine in response to a meal. Physiologically, it acts to reduce gastric motility and secretion. Although a unique receptor for OXM has yet to be identified, data from rodent and human studies suggest that systemic administration of OXM can reduce both food intake and body weight with some of these effects brought about by a suppression of the orexigenic hormone ghrelin (Chaudhri et al., 2006).

### The Endocrine Pancreas

#### Insulin

Insulin receptors are widely distributed in the brain with the highest concentrations found in the olfactory bulb, hippocampus, cerebral cortex, and the arcuate nucleus within the hypothalamus. The existence of neural circuits that appeared to respond to insulin and regulate food intake and adipose mass was reported many years before leptin was discovered (Schwartz et al., 1992). Despite this, neuron-specific loss of insulin receptors only has subtle effects on food intake and body weight (Bruning et al., 2000). Female (but not male) mice with disruption of the insulin receptor gene in the CNS become hyperphagic and overweight (10%–15% heavier than control), but this may be confounded by a reproductive endocrine phenotype.

The importance of a more localized population of insulin receptors, in particular those within the hypothalamus, has been further delineated. Obici et al. (2002) were able to significantly reduce hypothalamic insulin signaling by directly injecting into the third ventricle an antisense oligonucleotide designed to blunt the expression of insulin receptor in adjacent nuclei. This resulted in a rapid and significant hyperphagia, associated with an increase in the expression of both NPY and AgRP.

However, the role of insulin signaling at POMC neurons is not entirely clear. Choudhury et al. (2005) generated mice that lacked insulin receptor substrate 2 (IRS2) in POMC neurons. IRS2 is a major mediator of the metabolic effects of insulin. In contrast to the effects of leptin receptor loss from POMC neurons, mice lacking IRS2 from these neurons had no discernible phenotype, being neither hyperphagic nor obese, suggesting that insulin signaling in POMC neurons does not play a major role in energy homeostasis. Moreover, further complexity comes from data demonstrating that, in direct contrast to leptin, insulin actually hyperpolarizes POMC neurons and decreases the neuronal firing rate, indicating that the anorexigenic effects of insulin are unlikely to be mediated via POMC neurons (Plum et al., 2006).

After many decades of experiment and debate it is still difficult to place insulin's role in the normal physiology of energy homeostasis. Insulin undoubtedly has a major and nonredundant role in the control of the disposition of nutrients in the periphery. Although insulin clearly has appetite-suppressive effects when administered to the CNS, the physiological significance of such actions remains uncertain.

#### Pancreatic Polypeptide

Pancreatic polypeptide (PP) is released from cells found at the periphery of pancreatic islets, again in proportion to the amount of calories ingested with the effect of inhibiting gastric emptying. Whether PP has an additional physiological role in the control of food intake remains uncertain although infusions of PP mimicking the postprandial rise can suppress food intake in man (Chaudhri et al., 2006).

### Other Sites

Circulating levels of glucocorticoids derived from the adrenal gland are able to profoundly affect appetite (Dallman et al., 2004). Anorexia is a feature of the cortisol deficiency seen in primary adrenal failure, whereas an excess of glucocorticoids can cause hyperphagia. Glucocorticoids also have a significant impact upon the melanocortin system. Adrenalectomy reverses the obese phenotype in leptin-deficient *ob*/*ob* mice and by normalizing both the increase in AgRP expression and the decrease in POMC expression found in *ob*/*ob* mice restores hypothalamic melanocortin tone (Makimura et al., 2000). In addition, the orexigenic effect of the melanocortin antagonist AgRP is absent in adrenalectomized rats but restored with supplementation of glucocorticoids (Drazen et al., 2003).

Steroid hormones derived from the gonads have also been implicated in the modulation of eating behavior. Exciting recent data have shown that estradiol can reduce body weight by increasing excitatory synapses upon POMC soma. This increase in POMC neuronal activity results in both a reduction in energy intake and an increase in energy expenditure (Gao et al., 2007).

Thyroid hormones can influence feeding behavior, as tri-iodothyronine (T3), acting in the CNS, is able to increase food intake (Kong et al., 2004). However, this must be tempered by the fact that T3 induces a marked increase in basal metabolic rate, and thus any increase in food intake may be a compensatory response to this increase in energy expenditure.

Hepatocyte-derived C-reactive protein (CRP) has been correlated with adiposity and levels of plasma leptin. Chen et al. (2006) have reported that CRP binds leptin, that this interaction attenuates leptin action in vivo, and that leptin stimulates CRP production from hepatocytes. This suggests a possible vicious cycle in which the stimulation of CRP production by leptin leads to the production of greater quantities of a leptin antagonist, thereby being a potential mechanism contributing to leptin resistance seen in obese subjects. However, the relationship between CRP and leptin remains uncertain. For example, we have recently found that when leptin-naïve children with congenital leptin deficiency are treated with leptin there is no change in circulating CRP concentrations (Farooqi and O'Rahilly, 2007), and we have been unable to find any antagonism of leptin actions in mice co-treated with leptin and a 6-fold molar excess of highly purified CRP (Hutchinson et al., 2007).

### Summary

The genetic evidence for a critical role of leptin and its downstream anorexigenic pathways in the control of food intake is extremely strong and highly suggestive of a system with little redundancy. The severe and continued hyperphagia seen in rodents and humans when the leptin signaling system is disrupted, which persists in the face of extreme expansion of the adipose tissue mass, suggests that other adipocyte-derived signals play at most a subsidiary role in adipostatic control of food intake.

In contrast, the evidence for one particular gut-derived peptide having such a singular role in appetitive control is weaker, suggestive of a system in which there is a high degree of redundancy. However, this does not exclude a physiological role for such peptides. Instead, it suggests the presence of an integrated system in which adipocyte-derived signals provide tonic, long-term information to the brain about the state of nutrient stores, whereas a variety of signals triggered by ingestive status have important roles in influencing meal initiation and termination. Future experiments directed toward the simultaneous ablation of multiple putatively anorectic gut- and pancreas-derived peptides should help clarify the impact of the gut-brain axis in meal termination and initiation.

An improved understanding of the normal physiology of energy homeostasis and the endocrine control of food intake will have profound implications for the development of effective therapeutic and preventive strategies for human obesity. Contrary to popular misconception, obesity is not a new disease that has suddenly appeared as a result of a toxic environment. Human populations have always consisted of individuals with a broad range of adiposity, with those at the top end of the distribution suffering adverse health consequences. Recent changes in the availability and cost of palatable energy-dense food and the reduction in physical activity during work and recreation have undoubtedly contributed to an overall upward shift in the adiposity of entire populations. However, even in communities with substantial obesity there is still a high proportion of subjects who are lean. The heritability of adiposity is very high, and the most parsimonious explanation is that genetically based variation in the homeostatic pathways controlling energy balance determines the interindividual differences in susceptibility or resistance to an environment that fosters obesity. Such a model does not exclude the fact that body weight can be altered by voluntary efforts of human will but adds the important rider that the ease with which this can be achieved differs markedly between people and suggests that those differences are likely to be based on the variable intensity of inherent biological drives, not “moral fiber.” This conceptual approach to the undoubted health problem of obesity should encourage biomedical scientists to, as Friedman has put it, “make war on obesity, not the obese” (Friedman, 2003).

## References

[bib1] Alharbi K.K., Spanakis E., Tan K., Smith M.J., Aldahmesh M.A., O'Dell S.D., Sayer A.A., Lawlor D.A., Ebrahim S., Smith G.D. (2007). Prevalence and functionality of paucimorphic and private MC4R mutations in a large, unselected European British population, scanned by meltMADGE. Hum. Mutat..

[bib2] Alon T., Friedman J.M. (2006). Late-onset leanness in mice with targeted ablation of melanin concentrating hormone neurons. J. Neurosci..

[bib3] Balthasar N., Dalgaard L.T., Lee C.E., Yu J., Funahashi H., Williams T., Ferreira M., Tang V., McGovern R.A., Kenny C.D. (2005). Divergence of melanocortin pathways in the control of food intake and energy expenditure. Cell.

[bib4] Balthasar N., Coppari R., McMinn J., Liu S.M., Lee C.E., Tang V., Kenny C.D., McGovern R.A., Chua S.C., Elmquist J.K., Lowell B.B. (2004). Leptin receptor signaling in POMC neurons is required for normal body weight homeostasis. Neuron.

[bib5] Bates S.H., Myers M.G. (2003). The role of leptin receptor signaling in feeding and neuroendocrine function. Trends Endocrinol. Metab..

[bib6] Batterham R.L., Heffron H., Kapoor S., Chivers J.E., Chandarana K., Herzog H., Le Roux C.W., Thomas E.L., Bell J.D., Withers D.J. (2006). Critical role for peptide YY in protein-mediated satiation and body-weight regulation. Cell Metab..

[bib7] Bence K.K., Delibegovic M., Xue B., Gorgun C.Z., Hotamisligil G.S., Neel B.G., Kahn B.B. (2006). Neuronal PTP1B regulates body weight, adiposity and leptin action. Nat. Med..

[bib8] Biebermann H., Castaneda T.R., van Landeghem F., von Deimling A., Escher F., Brabant G., Hebebrand J., Hinney A., Tschop M.H., Gruters A., Krude H. (2006). A role for beta-melanocyte-stimulating hormone in human body-weight regulation. Cell Metab..

[bib9] Bouret S.G., Draper S.J., Simerly R.B. (2004). Trophic action of leptin on hypothalamic neurons that regulate feeding. Science.

[bib10] Bruning J.C., Gautam D., Burks D.J., Gillette J., Schubert M., Orban P.C., Klein R., Krone W., Muller-Wieland D., Kahn C.R. (2000). Role of brain insulin receptor in control of body weight and reproduction. Science.

[bib11] Chaudhri O., Small C., Bloom S. (2006). Gastrointestinal hormones regulating appetite. Philos. Trans. R. Soc. Lond. B Biol. Sci..

[bib12] Chen K., Li F., Li J., Cai H., Strom S., Bisello A., Kelley D.E., Friedman-Einat M., Skibinski G.A., McCrory M.A. (2006). Induction of leptin resistance through direct interaction of C-reactive protein with leptin. Nat. Med..

[bib13] Chida D., Osaka T., Hashimoto O., Iwakura Y. (2006). Combined interleukin-6 and interleukin-1 deficiency causes obesity in young mice. Diabetes.

[bib14] Choudhury A.I., Heffron H., Smith M.A., Al-Qassab H., Xu A.W., Selman C., Simmgen M., Clements M., Claret M., Maccoll G. (2005). The role of insulin receptor substrate 2 in hypothalamic and beta cell function. J. Clin. Invest..

[bib15] Coll A.P., Farooqi I.S., Challis B.G., Yeo G.S., O'Rahilly S. (2004). Proopiomelanocortin and energy balance: insights from human and murine genetics. J. Clin. Endocrinol. Metab..

[bib16] Cone R.D. (2005). Anatomy and regulation of the central melanocortin system. Nat. Neurosci..

[bib17] Cowley M.A., Smith R.G., Diano S., Tschop M., Pronchuk N., Grove K.L., Strasburger C.J., Bidlingmaier M., Esterman M., Heiman M.L. (2003). The distribution and mechanism of action of ghrelin in the CNS demonstrates a novel hypothalamic circuit regulating energy homeostasis. Neuron.

[bib18] Dallman M.F., la Fleur S.E., Pecoraro N.C., Gomez F., Houshyar H., Akana S.F. (2004). Minireview: glucocorticoids–food intake, abdominal obesity, and wealthy nations in 2004. Endocrinology.

[bib19] Dhillon H., Zigman J.M., Ye C., Lee C.E., McGovern R.A., Tang V., Kenny C.D., Christiansen L.M., White R.D., Edelstein E.A. (2006). Leptin directly activates SF1 neurons in the VMH, and this action by leptin is required for normal body-weight homeostasis. Neuron.

[bib20] Drazen D.L., Wortman M.D., Schwartz M.W., Clegg D.J., van Dijk G., Woods S.C., Seeley R.J. (2003). Adrenalectomy alters the sensitivity of the central nervous system melanocortin system. Diabetes.

[bib21] Drucker D.J. (2006). The biology of incretin hormones. Cell Metab..

[bib22] Fan W., Ellacott K.L., Halatchev I.G., Takahashi K., Yu P., Cone R.D. (2004). Cholecystokinin-mediated suppression of feeding involves the brainstem melanocortin system. Nat. Neurosci..

[bib23] Farooqi I.S., O'Rahilly S. (2007). Is leptin an important physiological regulator of CRP?. Nat. Med..

[bib24] Farooqi I.S., Matarese G., Lord G.M., Keogh J.M., Lawrence E., Agwu C., Sanna V., Jebb S.A., Perna F., Fontana S. (2002). Beneficial effects of leptin on obesity, T cell hyporesponsiveness, and neuroendocrine/metabolic dysfunction of human congenital leptin deficiency. J. Clin. Invest..

[bib25] Farooqi I.S., Keogh J.M., Yeo G.S., Lank E.J., Cheetham T., O'Rahilly S. (2003). Clinical spectrum of obesity and mutations in the melanocortin 4 receptor gene. N. Engl. J. Med..

[bib26] Farooqi I.S., Wangensteen T., Collins S., Kimber W., Matarese G., Keogh J.M., Lank E., Bottomley B., Lopez-Fernandez J., Ferraz-Amaro I. (2007). Clinical and molecular genetic spectrum of congenital deficiency of the leptin receptor. N. Engl. J. Med..

[bib27] Flier J.S. (2004). Obesity wars: molecular progress confronts an expanding epidemic. Cell.

[bib28] Flier J.S. (2006). AgRP in energy balance: Will the real AgRP please stand up?. Cell Metab..

[bib29] Friedman J.M. (2003). A war on obesity, not the obese. Science.

[bib30] Friedman J.M., Halaas J.L. (1998). Leptin and the regulation of body weight in mammals. Nature.

[bib31] Fulton S., Pissios P., Manchon R.P., Stiles L., Frank L., Pothos E.N., Maratos-Flier E., Flier J.S. (2006). Leptin regulation of the mesoaccumbens dopamine pathway. Neuron.

[bib32] Gao Q., Mezei G., Nie Y., Rao Y., Choi C.S., Bechmann I., Leranth C., Toran-Allerand D., Priest C.A., Roberts J.L. (2007). Anorectic estrogen mimics leptin's effect on the rewiring of melanocortin cells and Stat3 signaling in obese animals. Nat. Med..

[bib33] Gray J., Yeo G.S., Cox J.J., Morton J., Adlam A.L., Keogh J.M., Yanovski J.A., El Gharbawy A., Han J.C., Tung Y.C. (2006). Hyperphagia, severe obesity, impaired cognitive function, and hyperactivity associated with functional loss of one copy of the brain-derived neurotrophic factor (BDNF) gene. Diabetes.

[bib34] Grill H.J., Schwartz M.W., Kaplan J.M., Foxhall J.S., Breininger J., Baskin D.G. (2002). Evidence that the caudal brainstem is a target for the inhibitory effect of leptin on food intake. Endocrinology.

[bib35] Halatchev I.G., Cone R.D. (2005). Peripheral administration of PYY(3-36) produces conditioned taste aversion in mice. Cell Metab..

[bib36] Heisler L.K., Jobst E.E., Sutton G.M., Zhou L., Borok E., Thornton-Jones Z., Liu H.Y., Zigman J.M., Balthasar N., Kishi T. (2006). Serotonin reciprocally regulates melanocortin neurons to modulate food intake. Neuron.

[bib37] Hinney A., Bettecken T., Tarnow P., Brumm H., Reichwald K., Lichtner P., Scherag A., Nguyen T.T., Schlumberger P., Rief W. (2006). Prevalence, spectrum, and functional characterization of melanocortin-4 receptor gene mutations in a representative population-based sample and obese adults from Germany. J. Clin. Endocrinol. Metab..

[bib38] Holst B., Egerod K.L., Schild E., Vickers S.P., Cheetham S., Gerlach L.O., Storjohann L., Stidsen C.E., Jones R., Beck-Sickinger A.G., Schwartz T.W. (2007). GPR39 signaling is stimulated by zinc ions but not by obestatin. Endocrinology.

[bib39] Hommel J.D., Trinko R., Sears R.M., Georgescu D., Liu Z.W., Gao X.B., Thurmon J.J., Marinelli M., DiLeone R.J. (2006). Leptin receptor signaling in midbrain dopamine neurons regulates feeding. Neuron.

[bib40] Howard J.K., Cave B.J., Oksanen L.J., Tzameli I., Bjorbaek C., Flier J.S. (2004). Enhanced leptin sensitivity and attenuation of diet-induced obesity in mice with haploinsufficiency of Socs3. Nat. Med..

[bib41] Huo L., Grill H.J., Bjorbaek C. (2006). Divergent regulation of proopiomelanocortin neurons by leptin in the nucleus of the solitary tract and in the arcuate hypothalamic nucleus. Diabetes.

[bib42] Hutchinson W.L., Coll A.P., Gallimore J.R., Tennent G.A., Pepys M.B. (2007). Is leptin an important physiological regulator of CRP?. Nat. Med..

[bib43] Kennedy G.C. (1953). The role of depot fat in the hypothalamic control of food intake in the rat. Proc. R. Soc. Lond. B. Biol. Sci..

[bib44] Kievit P., Howard J.K., Badman M.K., Balthasar N., Coppari R., Mori H., Lee C.E., Elmquist J.K., Yoshimura A., Flier J.S. (2006). Enhanced leptin sensitivity and improved glucose homeostasis in mice lacking suppressor of cytokine signaling-3 in POMC-expressing cells. Cell Metab..

[bib45] King B.M. (2006). The rise, fall, and resurrection of the ventromedial hypothalamus in the regulation of feeding behavior and body weight. Physiol. Behav..

[bib46] Kokoeva M.V., Yin H., Flier J.S. (2005). Neurogenesis in the hypothalamus of adult mice: potential role in energy balance. Science.

[bib47] Kong W.M., Martin N.M., Smith K.L., Gardiner J.V., Connoley I.P., Stephens D.A., Dhillo W.S., Ghatei M.A., Small C.J., Bloom S.R. (2004). Triiodothyronine stimulates food intake via the hypothalamic ventromedial nucleus independent of changes in energy expenditure. Endocrinology.

[bib48] Kublaoui B.M., Holder J.L., Gemelli T., Zinn A.R. (2006). Sim1 haploinsufficiency impairs melanocortin-mediated anorexia and activation of paraventricular nucleus neurons. Mol. Endocrinol..

[bib49] Larsen L.H., Echwald S.M., Sorensen T.I., Andersen T., Wulff B.S., Pedersen O. (2005). Prevalence of mutations and functional analyses of melanocortin 4 receptor variants identified among 750 men with juvenile-onset obesity. J. Clin. Endocrinol. Metab..

[bib50] le Roux C.W., Aylwin S.J., Batterham R.L., Borg C.M., Coyle F., Prasad V., Shurey S., Ghatei M.A., Patel A.G., Bloom S.R. (2006). Gut hormone profiles following bariatric surgery favor an anorectic state, facilitate weight loss, and improve metabolic parameters. Ann. Surg..

[bib51] le Roux C.W., Batterham R.L., Aylwin S.J., Patterson M., Borg C.M., Wynne K.J., Kent A., Vincent R.P., Gardiner J., Ghatei M.A., Bloom S.R. (2006). Attenuated peptide YY release in obese subjects is associated with reduced satiety. Endocrinology.

[bib52] Lee M.J., Fried S.K. (2006). Multilevel regulation of leptin storage, turnover, and secretion by feeding and insulin in rat adipose tissue. J. Lipid Res..

[bib53] Lee Y.S., Challis B.G., Thompson D.A., Yeo G.S., Keogh J.M., Madonna M.E., Wraight V., Sims M., Vatin V., Meyre D. (2006). A POMC variant implicates beta-melanocyte-stimulating hormone in the control of human energy balance. Cell Metab..

[bib54] Licinio J., Caglayan S., Ozata M., Yildiz B.O., de Miranda P.B., O'Kirwan F., Whitby R., Liang L., Cohen P., Bhasin S. (2004). Phenotypic effects of leptin replacement on morbid obesity, diabetes mellitus, hypogonadism, and behavior in leptin-deficient adults. Proc. Natl. Acad. Sci. USA.

[bib55] Maeda N., Shimomura I., Kishida K., Nishizawa H., Matsuda M., Nagaretani H., Furuyama N., Kondo H., Takahashi M., Arita Y. (2002). Diet-induced insulin resistance in mice lacking adiponectin/ACRP30. Nat. Med..

[bib56] Makimura H., Mizuno T.M., Roberts J., Silverstein J., Beasley J., Mobbs C.V. (2000). Adrenalectomy reverses obese phenotype and restores hypothalamic melanocortin tone in leptin-deficient ob/ob mice. Diabetes.

[bib57] Michaud J.L., Boucher F., Melnyk A., Gauthier F., Goshu E., Levy E., Mitchell G.A., Himms-Hagen J., Fan C.M. (2001). Sim1 haploinsufficiency causes hyperphagia, obesity and reduction of the paraventricular nucleus of the hypothalamus. Hum. Mol. Genet..

[bib58] Mori H., Hanada R., Hanada T., Aki D., Mashima R., Nishinakamura H., Torisu T., Chien K.R., Yasukawa H., Yoshimura A. (2004). Socs3 deficiency in the brain elevates leptin sensitivity and confers resistance to diet-induced obesity. Nat. Med..

[bib59] Nakagawa T., Tsuchida A., Itakura Y., Nonomura T., Ono M., Hirota F., Inoue T., Nakayama C., Taiji M., Noguchi H. (2000). Brain-derived neurotrophic factor regulates glucose metabolism by modulating energy balance in diabetic mice. Diabetes.

[bib60] Nawrocki A.R., Rajala M.W., Tomas E., Pajvani U.B., Saha A.K., Trumbauer M.E., Pang Z., Chen A.S., Ruderman N.B., Chen H. (2006). Mice lacking adiponectin show decreased hepatic insulin sensitivity and reduced responsiveness to peroxisome proliferator-activated receptor gamma agonists. J. Biol. Chem..

[bib61] Netea M.G., Joosten L.A., Lewis E., Jensen D.R., Voshol P.J., Kullberg B.J., Tack C.J., van Krieken H., Kim S.H., Stalenhoef A.F. (2006). Deficiency of interleukin-18 in mice leads to hyperphagia, obesity and insulin resistance. Nat. Med..

[bib62] Nogueiras R., Pfluger P., Tovar S., Arnold M., Mitchell S., Morris A., Perez-Tilve D., Vazquez M.J., Wiedmer P., Castaneda T.R. (2007). Effects of obestatin on energy balance and growth hormone secretion in rodents. Endocrinology.

[bib63] Obici S., Feng Z., Karkanias G., Baskin D.G., Rossetti L. (2002). Decreasing hypothalamic insulin receptors causes hyperphagia and insulin resistance in rats. Nat. Neurosci..

[bib64] Oral E.A., Simha V., Ruiz E., Andewelt A., Premkumar A., Snell P., Wagner A.J., DePaoli A.M., Reitman M.L., Taylor S.I. (2002). Leptin-replacement therapy for lipodystrophy. N. Engl. J. Med..

[bib65] Pinto S., Roseberry A.G., Liu H., Diano S., Shanabrough M., Cai X., Friedman J.M., Horvath T.L. (2004). Rapid rewiring of arcuate nucleus feeding circuits by leptin. Science.

[bib66] Plum L., Ma X., Hampel B., Balthasar N., Coppari R., Munzberg H., Shanabrough M., Burdakov D., Rother E., Janoschek R. (2006). Enhanced PIP3 signaling in POMC neurons causes KATP channel activation and leads to diet-sensitive obesity. J. Clin. Invest..

[bib67] Ricci M.R., Lee M.J., Russell C.D., Wang Y., Sullivan S., Schneider S.H., Brolin R.E., Fried S.K. (2005). Isoproterenol decreases leptin release from rat and human adipose tissue through posttranscriptional mechanisms. Am. J. Physiol. Endocrinol. Metab..

[bib68] Rios M., Fan G., Fekete C., Kelly J., Bates B., Kuehn R., Lechan R.M., Jaenisch R. (2001). Conditional deletion of brain-derived neurotrophic factor in the postnatal brain leads to obesity and hyperactivity. Mol. Endocrinol..

[bib69] Sakurai T., Amemiya A., Ishii M., Matsuzaki I., Chemelli R.M., Tanaka H., Williams S.C., Richardson J.A., Kozlowski G.P., Wilson S. (1998). Orexins and orexin receptors: a family of hypothalamic neuropeptides and G protein-coupled receptors that regulate feeding behavior. Cell.

[bib70] Saper C.B., Scammell T.E., Lu J. (2005). Hypothalamic regulation of sleep and circadian rhythms. Nature.

[bib71] Schwartz M.W., Figlewicz D.P., Baskin D.G., Woods S.C., Porte D. (1992). Insulin in the brain: a hormonal regulator of energy balance. Endocr. Rev..

[bib72] Shklyaev S., Aslanidi G., Tennant M., Prima V., Kohlbrenner E., Kroutov V., Campbell-Thompson M., Crawford J., Shek E.W., Scarpace P.J., Zolotukhin S. (2003). Sustained peripheral expression of transgene adiponectin offsets the development of diet-induced obesity in rats. Proc. Natl. Acad. Sci. USA.

[bib73] Sun Y., Ahmed S., Smith R.G. (2003). Deletion of ghrelin impairs neither growth nor appetite. Mol. Cell. Biol..

[bib74] Sun Y., Wang P., Zheng H., Smith R.G. (2004). Ghrelin stimulation of growth hormone release and appetite is mediated through the growth hormone secretagogue receptor. Proc. Natl. Acad. Sci. USA.

[bib75] Theander-Carrillo C., Wiedmer P., Cettour-Rose P., Nogueiras R., Perez-Tilve D., Pfluger P., Castaneda T.R., Muzzin P., Schurmann A., Szanto I. (2006). Ghrelin action in the brain controls adipocyte metabolism. J. Clin. Invest..

[bib76] Trujillo M.E., Scherer P.E. (2005). Adiponectin–journey from an adipocyte secretory protein to biomarker of the metabolic syndrome. J. Intern. Med..

[bib77] Tschop M., Castaneda T.R., Joost H.G., Thone-Reineke C., Ortmann S., Klaus S., Hagan M.M., Chandler P.C., Oswald K.D., Benoit S.C. (2004). Physiology: does gut hormone PYY3-36 decrease food intake in rodents?. Nature.

[bib78] Wallenius V., Wallenius K., Ahren B., Rudling M., Carlsten H., Dickson S.L., Ohlsson C., Jansson J.O. (2002). Interleukin-6-deficient mice develop mature-onset obesity. Nat. Med..

[bib79] Welt C.K., Chan J.L., Bullen J., Murphy R., Smith P., DePaoli A.M., Karalis A., Mantzoros C.S. (2004). Recombinant human leptin in women with hypothalamic amenorrhea. N. Engl. J. Med..

[bib80] Williams D.L., Cummings D.E. (2005). Regulation of ghrelin in physiologic and pathophysiologic states. J. Nutr..

[bib81] Wortley K.E., Anderson K.D., Garcia K., Murray J.D., Malinova L., Liu R., Moncrieffe M., Thabet K., Cox H.J., Yancopoulos G.D. (2004). Genetic deletion of ghrelin does not decrease food intake but influences metabolic fuel preference. Proc. Natl. Acad. Sci. USA.

[bib82] Wortley K.E., Anderson K.D., Yasenchak J., Murphy A., Valenzuela D., Diano S., Yancopoulos G.D., Wiegand S.J., Sleeman M.W. (2005). Agouti-related protein-deficient mice display an age-related lean phenotype. Cell Metab..

[bib83] Wortley K.E., del Rincon J.P., Murray J.D., Garcia K., Iida K., Thorner M.O., Sleeman M.W. (2005). Absence of ghrelin protects against early-onset obesity. J. Clin. Invest..

[bib84] Xu B., Goulding E.H., Zang K., Cepoi D., Cone R.D., Jones K.R., Tecott L.H., Reichardt L.F. (2003). Brain-derived neurotrophic factor regulates energy balance downstream of melanocortin-4 receptor. Nat. Neurosci..

[bib85] Yamanaka A., Beuckmann C.T., Willie J.T., Hara J., Tsujino N., Mieda M., Tominaga M., Yagami K., Sugiyama F., Goto K. (2003). Hypothalamic orexin neurons regulate arousal according to energy balance in mice. Neuron.

[bib86] Yeo G.S., Connie Hung C.C., Rochford J., Keogh J., Gray J., Sivaramakrishnan S., O'Rahilly S., Farooqi I.S. (2004). A de novo mutation affecting human TrkB associated with severe obesity and developmental delay. Nat. Neurosci..

[bib87] Zhang J.V., Ren P.G., Avsian-Kretchmer O., Luo C.W., Rauch R., Klein C., Hsueh A.J. (2005). Obestatin, a peptide encoded by the ghrelin gene, opposes ghrelin's effects on food intake. Science.

[bib88] Zhang Y., Proenca R., Maffei M., Barone M., Leopold L., Friedman J.M. (1994). Positional cloning of the mouse obese gene and its human homologue. Nature.

[bib89] Zigman J.M., Nakano Y., Coppari R., Balthasar N., Marcus J.N., Lee C.E., Jones J.E., Deysher A.E., Waxman A.R., White R.D. (2005). Mice lacking ghrelin receptors resist the development of diet-induced obesity. J. Clin. Invest..

[bib90] Zorrilla E.P., Iwasaki S., Moss J.A., Chang J., Otsuji J., Inoue K., Meijler M.M., Janda K.D. (2006). From the cover: Vaccination against weight gain. Proc. Natl. Acad. Sci. USA.

